# The safety and effectiveness of lumbar transforaminal injection of sterile amniotic fluid filtrate compared to steroid for lumbosacral radicular pain due to spinal stenosis: a phase I/II double-blinded randomized trial

**DOI:** 10.1093/pm/pnag048

**Published:** 2026-03-31

**Authors:** Zachary L McCormick, Hasan Sen, Amanda N Cooper, Aaron M Conger, Alexandra E Fogarty, Allison Glinka Przybysz, Taylor Burnham, Richard Kendall, Graham Wagner, Pamela A Hansen, Jan Pierce, Brooke Hashimoto, Daniel M Cushman, Brook I Martin

**Affiliations:** Department of Physical Medicine and Rehabilitation, University of Utah, Salt Lake City, UT, United States; Department of Physical Medicine and Rehabilitation, University of Utah, Salt Lake City, UT, United States; Department of Physical Medicine and Rehabilitation, University of Utah, Salt Lake City, UT, United States; Department of Physical Medicine and Rehabilitation, University of Utah, Salt Lake City, UT, United States; Department of Physical Medicine and Rehabilitation, University of Utah, Salt Lake City, UT, United States; Department of Physical Medicine and Rehabilitation, University of Utah, Salt Lake City, UT, United States; Department of Physical Medicine and Rehabilitation, University of Utah, Salt Lake City, UT, United States; Division of Physical Medicine and Rehabilitation, University of Calgary, Calgary, AB, Canada; Vivo Cura Health, Calgary, AB, Canada; Department of Physical Medicine and Rehabilitation, University of Utah, Salt Lake City, UT, United States; Department of Physical Medicine and Rehabilitation, University of Utah, Salt Lake City, UT, United States; Department of Physical Medicine and Rehabilitation, University of Utah, Salt Lake City, UT, United States; Cell Therapy and Regenerative Medicine Program, University of Utah School of Medicine, Salt Lake City, UT, United States; Cell Therapy and Regenerative Medicine Program, University of Utah School of Medicine, Salt Lake City, UT, United States; Department of Physical Medicine and Rehabilitation, University of Utah, Salt Lake City, UT, United States; Department of Orthopedics, University of Utah, Salt Lake City, UT, United States

**Keywords:** amniotic fluid, epidural injection, spinal stenosis, steroid

## Abstract

**Background:**

Radicular symptoms from lumbosacral spinal stenosis (LSS) are commonly managed with conservative measures, including transforaminal epidural steroid injection (TFESI). However, repeated use may cause systemic side effects in some patients. Human amniotic fluid filtrate (hAF), containing anti-inflammatory, neuroprotective, and regenerative factors, has shown therapeutic potential in pre-clinical models and other medical conditions.

**Objectives:**

Compare the safety and effectiveness of transforaminal epidural injections with hAF versus dexamethasone for treating radicular pain from LSS.

**Design:**

Double-blinded, prospective, randomized comparative trial.

**Methods:**

Fifty-eight participants were included, with outcomes assessed at 3 weeks, 6 weeks, 3 months (primary endpoint), and 6 months. Primary outcomes were adverse events (AEs) and proportions of participants reporting ≥50% numerical pain rating scale (NPRS) back and leg pain reductions at 3 months. Secondary outcomes included Oswestry Disability Index (ODI), Swiss Spinal Stenosis Questionnaire (SSSQ), and PROMIS Physical Health Summary (PHS). Linear mixed-effects models evaluated between-group differences while accounting for repeated measures.

**Results:**

At 3 months, a significantly greater proportion of participants receiving dexamethasone achieved ≥50% reductions in back pain (45.8% [95%CI = 27.9%-64.9%] vs. 15.0% [95%CI = 5.2%-36.0%]; *P *= .050) and leg pain (60.0% [95%CI = 40.7%-76.6%] vs. 25.0% [95%CI = 11.2%-46.9%]; *P *= .034) compared with hAF. Secondary outcome trends similarly favored dexamethasone at 3 months. Mixed-effects models showed greater improvements to 6-week pain and function scores with dexamethasone relative to hAF. Group AE rates were comparable, and no serious intervention-related AEs occurred.

**Conclusion:**

Dexamethasone demonstrated significantly greater short- and intermediate-term benefits over hAF across multiple clinical outcome domains for radicular pain due to LSS, prompting early trial discontinuation.

**Trial Registration:**

ClinicalTrials.gov (NCT04537026); September 3, 2020.

## Teaser

This double-blinded randomized trial compared transforaminal epidural injections of human amniotic fluid filtrate (hAF) and dexamethasone in 58 participants with radicular pain due to lumbosacral spinal stenosis. At 3 months (the primary endpoint), significantly more dexamethasone-treated patients achieved ≥50% reductions in back and leg pain. Secondary outcomes and mixed-effects models similarly favored dexamethasone. Adverse event rates were comparable, with no serious treatment-related events. Dexamethasone showed superior short- and intermediate-term benefits, leading to early trial discontinuation.

## Introduction

Lumbar spinal stenosis (LSS) is a prevalent condition that contributes significantly to chronic low back and leg pain symptoms.^1^ LSS develops when hypertrophic changes of the facet joints, intervertebral disc height reduction, and ligamentum flavum buckling lead to narrowing of the spinal canal, lateral recesses, and/or foramina. This may result in fixed or intermittent compression of one or more lumbosacral nerve roots, causing lumbosacral radicular pain and/or neurogenic claudication.^2^ With an estimated prevalence of 11%-39%,^3^ symptomatic LSS is one of the leading causes of pain, disability, and healthcare utilization in the United States and globally.^4^

In the absence of a progressive neurological deficit, initial management of lumbosacral radicular pain due to LSS typically involves conservative treatments, including physical therapy, activity modification, and oral analgesic or neuropathic medications.^5^ Physical therapy focusing on flexibility, strengthening, and aerobic conditioning may improve function and reduce pain in some patients, but the quality of evidence is moderate and benefits are often modest and not sustained long-term.^6^ Activity modification is commonly recommended, though high-quality evidence for its effectiveness is lacking.^7^ Oral analgesics, including NSAIDs and neuropathic agents like gabapentinoids, can offer symptomatic relief but many are off-label, limited by side effects and generally do not provide lasting functional improvement, leading many patients to pursue further treatments.^8^^,^^9^

When conservative management fails or is not tolerated, epidural steroid injections (ESIs) are commonly used as a second-line treatment.^10–17^ ESIs are widely administered, with over 25% of all epidural injections in the Medicare population performed for spinal stenosis-related pain, amounting to more than 500 000 injections annually.^18^^,^^19^ When performed according to best practice standards,^20^ ESIs are generally safe.^21–23^ However, ESIs are associated with a range of systemic side effects, particularly with repeated use, including osteoporosis, adrenal suppression, hypertension, cataracts, gastrointestinal bleeding, and immune dysfunction.^24^ Short-term adverse effects such as flushing, headache, insomnia, transient hypertension, and hyperglycemia in diabetic patients are also common.^25^ There are conflicting reports regarding the effectiveness of transforaminal epidural steroid injections (TFESIs) for LSS-related radicular pain, leading to ongoing debate and controversy.^11^^,^^26–29^ Unlike disc herniations, which often resolve spontaneously, LSS tends to be a chronic and progressive condition, making effective long-term management strategies crucial.^30^^,^^31^ Many patients fail conservative treatment yet wish to avoid or are ineligible for surgery; as such, there is a critical need for safer and more effective alternatives to epidural corticosteroids.

Given its neuroprotective, anti-inflammatory, and regenerative properties, human amniotic fluid (hAF) is an attractive biologic therapy for potentially addressing these challenges. hAF naturally functions as a supportive and protective medium during fetal development, containing a rich source of nutrients, cytokines, and growth factors essential for tissue repair and regeneration.^32^ Studies suggest that hAF has immunomodulatory and growth-promoting properties, with evidence supporting its ability to enhance nerve regeneration,^33^ reduce inflammation,^34^ and promote wound healing.^35^ The presence of key bioactive factors such as epidermal growth factor (EGF), insulin-like growth factor (IGF-1), fibroblast growth factor (FGF), hepatocyte growth factor (HGF), and transforming growth factor-alpha (TGF-α) further supports its therapeutic potential.^36^^,^^37^ Additionally, hAF contains hyaluronic acid, which has been shown to minimize scarring and adhesions while promoting tissue repair.^33^ Since LSS is associated with chronic inflammation, degenerative changes, and neural irritation and/or compression, hAF may offer clinical benefits beyond pain relief by addressing underlying pathophysiological mechanisms.

This FDA-regulated Investigational New Drug (IND) study evaluated the safety and effectiveness of transforaminal epidural injection of human amniotic fluid (hAF) compared with dexamethasone for treating radicular pain secondary to lumbar spinal stenosis.

## Methods

### Study design

This IRB-approved (IRB_131761), double-blinded, randomized, prospective comparative trial was conducted at a single tertiary academic center within the University of Utah Department of Physical Medicine and Rehabilitation (PM&R). The study was supported by the University of Utah’s Cell Therapy and Regenerative Medicine program, with additional research grant support from the Skaggs Foundation for Research. The trial was registered with ClinicalTrials.gov (NCT04537026), and FDA authorization for study drug use in a human research trial was obtained (IND #26950).

Eligibility, screening, randomization, baseline information, MRI imaging results, safety reports, and participant-reported outcome data were housed in a web-enabled, third-party database (Statix LLC). Data systems, procedures, and policies comply with the Health Insurance Portability and Accountability Act, the Code of Federal Regulations Title 21 Part 11, the Federal Information Security Modernization Act, and computing principles of minimum necessity, separation of duties, and least privilege.^38^

### Patient selection and enrollment

Participants were recruited and treated between August 2021 and January 2025. All patients provided written informed consent before undergoing any study intervention. Eligibility was determined based on the criteria outlined in Table 1. Potential study candidates were identified during PM&R outpatient clinic visits. Patients were then approached by clinical research staff for enrollment and eligibility screening, with study investigators ultimately responsible for the final decision to include patients in the study. Prior to study enrollment, all patients were required to have a confirmed diagnosis of LSS based on both clinical symptoms and imaging findings according to the Boden characterization.^39^ Patients with mild to severe foraminal or subarticular zone stenosis and/or mild to moderate central canal spinal stenosis that correlated with their clinical symptoms and physical examination were considered eligible for inclusion.

**Table 1:** Participant eligibility criteria.

Inclusion criteria	Exclusion criteria
Age 18 or older NPRS ≥ 4 for unilateral or bilateral leg, with leg pain ≥ back pain Radicular distribution of leg pain based on history and correlation with advanced imaging Pain resistant to conservative therapy (ie, oral steroid, nonsteroidal anti-inflammatory drugs (NSAIDS), opioids, muscle relaxants, physical therapy, chiropractic, or other non-invasive care) for at least 3 weeks Mild to severe lumbar foraminal or subarticular zone stenosis, and/or mild to moderate central canal stenosis on MRI or CT according to radiologic criteria established by Boden^39^ Ability to provide informed consent, read and complete assessment questionnaires	Lumbar ESI in the last 90 days Unwilling or unable to comply with study procedures or provide consent Systemic infection or local infection over planned injection site Bleeding disorder or currently using anticoagulants or anti-platelet medications Intrinsic spinal cord lesion Central neurological, cerebrovascular, demyelinating, or muscular disease Vascular, pulmonary, or coronary artery disease that limits ambulation, including recent myocardial infarction within the last 6 months Allergy to any medication used for injection procedures Pregnant, breastfeeding, or planning to become pregnant during the study or unwilling to use effective birth control while participating in the study Cognitive deficit or motor neuron disease Spinal instability requiring surgery History of lumbar spinal fusion surgery Metastatic cancer Concordant pain with internal rotation of the hip (or known hip joint pathology)

Repeat/crossover injections	

Requested by participant and approved by principal investigator <50% NPRS reduction in *either* back *or* leg pain No possibly or probably related SAEs occurred with previous injection No severe hypersensitivity/acute inflammatory reaction requiring invasive intervention with previous injection	

Abbreviations: ESI, epidural steroid injection; NPRS, numerical pain rating scale; SAE, serious adverse event.

### Randomization and blinding

Participants were randomly assigned to receive transforaminal epidural injections with either hAF or dexamethasone in a 1:1 ratio using computer-generated block randomization stratified by treating physician. Block sizes were randomly varied, with even numbers ranging from 4 to 12, to minimize the potential for research staff to predict treatment assignment. Randomization was performed on the day of the procedure while the participant waited in the intake room. Only the unblinded research coordinator and the procedure nurse, who prepared the drug in the procedure room, were aware of the treatment assignment.

The participants, other research staff, treating physician, and follow-up interviewers were blinded to the treatment assignments throughout the entire study period. The prepared study medications were indistinguishable from one another. Although dexamethasone and hAF solutions are relatively translucent and odorless, both treatments were prepared with a 3-mL syringe covered with opaque stickers to conceal the injectate. The blinded treating physicians were allowed unblinding if a safety concern emerged; however, this did not occur. As evidence of concealment success, participants were asked during follow-up whether they knew or suspected which treatment they had received.

### Study interventions

All procedures were performed by four PM&R physicians with subspecialty fellowship training in Pain Medicine or Interventional Spine and Musculoskeletal Medicine. Participants received fluoroscopically-guided transforaminal epidural injections with either 1 mL dexamethasone sodium phosphate (10 mg/mL) combined with 2 mL of sterile water (control) or a 3-mL injection of hAF, depending on randomization. Each participant was allowed up to two total injections during the first three months of the study period (an index study injection according to randomization and then a repeat injection with the same injectate). A repeat index injection was offered to participants within two weeks to three months of the baseline injection. Participants had the option to cross-over to the alternative treatment at the three-month mark after their baseline injection. In the case of election for cross-over, participants who met the eligibility criteria outlined in Table 1 were permitted up to two additional injections (cross-over injection and repeat injection with the same cross-over injectate) between three and six months after the baseline injection. Participants could opt to receive the repeat cross-over injection within two weeks to three months of their initial cross-over injection.

After the first five participants with unilateral symptoms had been enrolled and underwent unilateral treatments, the data safety monitoring board (DSMB) allowed enrollment and treatment of participants with bilateral symptoms after finding no concerning safety events associated with the 3-mL injection volume. This safety check point was requested by the FDA. In the case of participants with bilateral symptoms, study interventions were identical, except the allocated injectate was administered on both the left and the right. For example, a participant with bilateral L5 radicular symptoms would have received bilateral L5-S1 transforaminal injections that included 3 mL of the allocated injectate on each side.

Lumbar transforaminal epidural injections were performed according to current best practice standards.^20^ The lumbosacral epidural injection procedure began by positioning the participant prone on a fluoroscopy table. After a pre-procedure time-out was performed, using fluoroscopic guidance, 1-3 mL of 1% lidocaine was injected into the skin and subcutaneous tissue to provide anesthesia over the site of planned entry to the neural foramen. A 22- or 25-g spinal needle (3.5ʺ-7ʺ) was used to access the epidural space using the sub-pedicular or infraneural transforaminal approach, depending on individual anatomy at the discretion of the treating physician, consistent with clinical practice guidelines.^20^ Needle tip position was confirmed using anterior-posterior and lateral fluoroscopic views as well as with injection of 0.5-3 mL of iodinated contrast medium (Omnipaque [Iohexol] or Isovue [Iopamidol]) during live fluoroscopy to confirm epidural flow of contrast and to rule out an intravascular injection. Then, depending on treatment allocation, either 3 mL of hAF or a 3-mL solution containing 1 mL of dexamethasone sodium phosphate (10 mg/mL) combined with 2 mL of sterile water was injected through the spinal needle for unilateral symptoms, for a total injection volume of 3 mL in both groups. Participants with bilateral symptoms underwent an identical procedure on the left and right sides at the same spinal level with a total of 6 mL of injection volume administered, split equally between sides.

Usual care co-interventions were allowed 3 months after the index study injection, including but not limited to physical therapy, chiropractic care, additional epidural injections, and spinal surgery.

### Outcome assessment

The primary outcomes for this study were (1) the number of adverse events associated with the injections (hAF versus dexamethasone), assessed through a standardized survey and review of systems, and (2) the percentage of participants reporting ≥50% NPRS improvement at 3 months, with back and leg pain assessed separately.^40^

Continuous secondary outcomes included mean score changes on Numeric Pain Rating Scale (NPRS; back and leg pain assessed separately), Oswestry Disability Index (ODI), Patient-Reported Outcomes Measurement Information System (PROMIS) Physical Health Summary (PHS) and Mental Health Summary (MHS), and Swiss Spinal Stenosis Questionnaire (SSSQ). Categorical secondary outcomes included between-group differences in the proportion of participants who elected to cross over to the alternative injection at 3 months, as well as repeat and cross-over injection rates. Responder analyses compared the proportions of participants in each group reporting (1) ≥30% improvement in ODI,^41^ (2) ≥30% improvement in SSSQ symptom severity, pain, and neuroischemic scores,^41^ (3) at least 5-point improvement in PROMIS PHS and MHS scores,^42^ and scores of 5-6 (corresponding to “better” or “a great deal better”) on Patient Global Perception of Change (PGIC). Adverse events associated with the procedure were recorded and categorized according to severity, expectedness, and relatedness to the study intervention (Table S1).

### Data analysis

Demographic, clinical, and MRI data were initially summarized as means for continuous measures with between-group comparisons based on t-tests, and as proportions for categorical variables with comparisons based on chi-square tests (or Fisher’s exact test in cases where expected frequencies were ≤5). A 95% CI was calculated for select statistics.

Multivariable random-effects linear regression models were used to separately estimate the treatment effect differences for hAF relative to dexamethasone for each continuous outcome over time, with measures at 3 weeks, 6 weeks, 3 months, and 6 months. Because a greater proportion of patients in the dexamethasone group reported pain lasting >5 years, we included pain duration as a categorical fixed variable in the models, along with original treatment assignment, timepoint, and the interaction between treatment assignment and timepoint. A random intercept for each subject and random slope for timepoint, with an unstructured covariance, adjusted estimates for clustering of measurements over time nested within each participant. Primary inference of treatment effect difference was based on interpretation of the beta coefficient for the hAF x timepoint interaction term (representing the additional mean score change from baseline in the hAF group compared to the dexamethasone group at each follow-up timepoint) and its significance. The total number of adverse events (AEs) and the proportion of participants who experienced AEs were compared between treatment groups using chi-square/Fisher’s exact tests. Comparisons of the mean number of AEs per participant were based on t-tests. All analyses were performed using Stata-MP version 18.5 (College Station, TX) with a two-sided alpha level of 0.05.

### Power analysis

We powered our study for 112 total participants (*n *= 56 per group) based on 1:1 randomization and the primary outcome measure of the proportion of participants with ≥50% pain improvement. The highest quality study to date demonstrated a successful response of 38% when using dexamethasone epidural injection for the indication of leg pain due to LSS.^26^ Because no studies of epidural hAF injection were available at the time the study protocol was created, a conservative estimate of 65% was used based on response rates from pilot data, which ranged from 70%-75%. Using these parameters, 50 participants in each group would provide 80% power to detect a significant difference between groups using an alpha value of 0.05.^43^ However, we increased our enrollment goal to 56 participants per group to account for an estimated attrition rate of 10%.

## Results

### Study population

**Figure 1:**
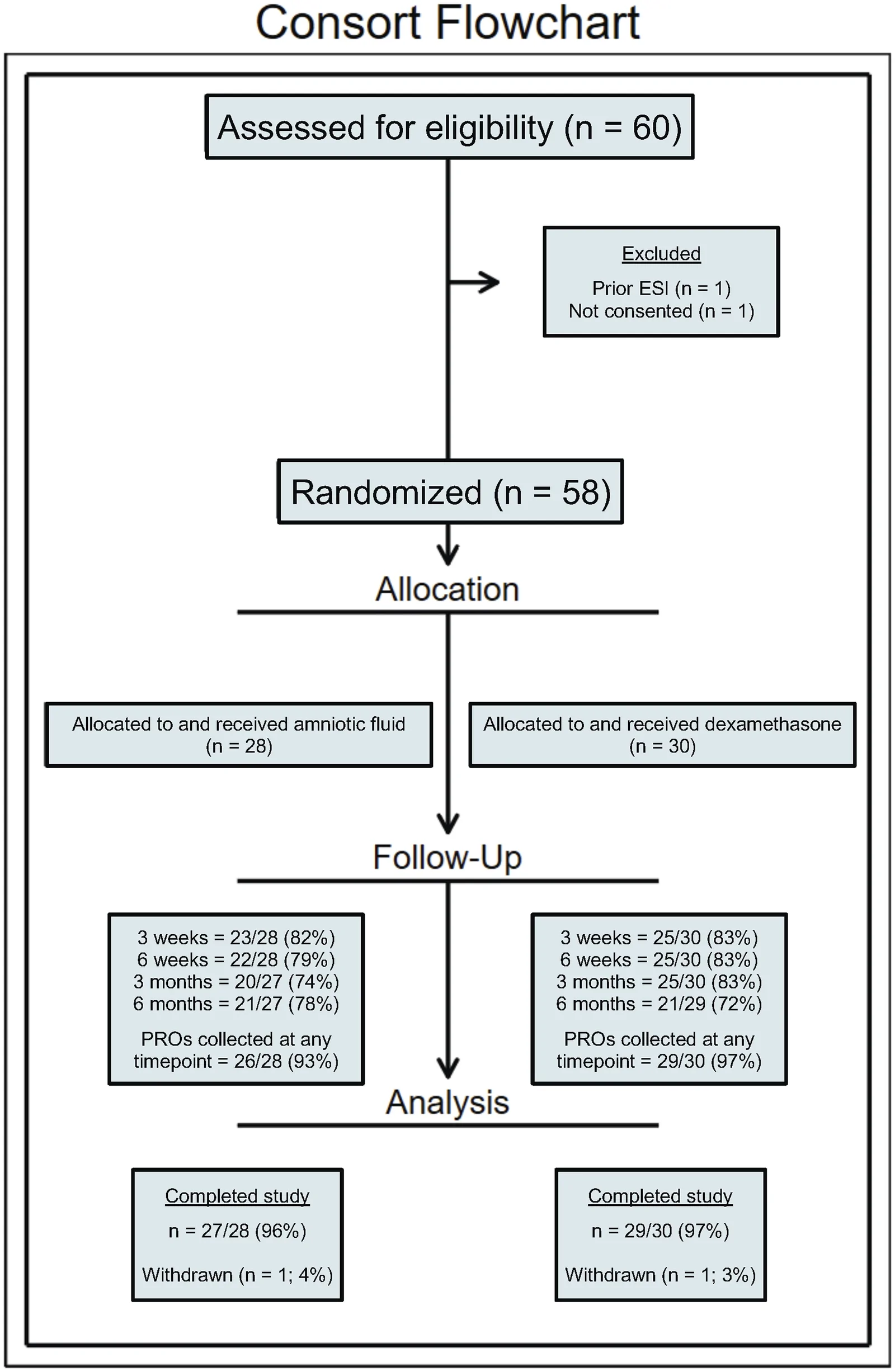
Study flow diagram. ESI = epidural steroid injection; PRO = patient-reported outcome.

Sixty patients with radicular pain due to LSS were assessed for study eligibility. A total of 58 participants were randomized, with 28 and 30 participants allocated to the hAF and dexamethasone treatment groups, respectively. All 58 participants received injections corresponding to their original treatment assignment. Two participants (*n *= 1 per group) withdrew prior to completing the final 6-month follow-up visit. A CONSORT flow diagram outlining participant progression through the study is presented in Figure 1.

Following an interim analysis, the Data Safety Monitoring Board (DSMB) recommended ending study enrollment early, with final outcomes collected at 6 months instead of the original study endpoint of 2 years. This recommendation was due to the dexamethasone group demonstrating significantly superior outcomes compared to the hAF group according to patient-reported outcomes related to pain reduction and a significantly lower rate of repeat injection in the dexamethasone (standard care) group compared to the hAF group. The additional sample size needed to overcome the observed differences made study continuation infeasible. Notably, the DSMB had no safety concerns, and the recommendation was not based on safety differences between the two study groups. As such, enrollment was stopped at 58 participants with a minimum of 6-month surveillance on all subjects. Assessment of outcomes based on this sample is subsequently presented.

### Baseline demographics, blinding assessment, and crossover/repeat injections

Participant demographics and clinical characteristics at baseline are summarized in Tables 2 and 3. All baseline characteristics were similar between treatment groups, with the exception of pain duration. A significantly greater proportion of participants in the dexamethasone group reported a pain duration of >5 years compared to the hAF group (*P *= .045).

**Table 2:** Participant demographics and clinical characteristics.

	Original treatment assignment		
Variable	hAF (*n *= 28)	Dexamethasone (*n *= 30)	*P*
Gender, *n* (%)			
Female	14 (50.0)	21 (70.0)	.120^a^
Male	14 (50.0)	9 (30.0)	
Race, *n* (%)			
White	24 (92.3)	28 (96.6)	.732
Black	1 (3.8)	0 (0.0)	
Other	1 (3.8)	1 (3.4)	
*Unknown*	*2*	*1*	
Hispanic, *n* (%)			.344
No	25 (89.3)	29 (96.7)	
Yes	3 (10.7)	1 (3.3)	
Work status, *n* (%)			.759
Working	14 (50.0)	14 (46.7)	
Sick leave or disability	3 (10.7)	2 (6.7)	
Student, retired, homemaker	11 (39.3)	12 (40.0)	
Not working	0 (0.0)	2 (6.7)	
Education, *n* (%)			.792
No high school diploma	1 (3.6)	0 (0.0)	
High school graduate or GED	2 (7.1)	1 (3.3)	
Some college, no degree	7 (25.0)	6 (20.0)	
Associate degree	2 (7.1)	2 (6.7)	
Bachelor’s degree	6 (21.4)	11 (36.7)	
Master’s degree (MA, MS, M Eng, Med, MBA)	9 (32.1)	7 (23.3)	
Professional school degree (MD, DDS, DVM, JD)	1 (3.6)	2 (6.7)	
Doctoral degree (PhD, EdD)	0 (0.0)	1 (3.3)	
Smoking status, *n* (%)			.504
Non smoker	23 (82.1)	27 (90.0)	
Current smoker	2 (7.1)	0 (0.0)	
Former smoker	3 (10.7)	3 (10.0)	
Prior low back surgery, *n* (%)			.860
None	22 (78.6)	25 (83.3)	
Yes, one operation	5 (17.9)	4 (13.3)	
Yes, more than one operation	1 (3.6)	1 (3.3)	
Pain frequency, *n* (%)			.491
Every day or nearly every day	25 (89.3)	23 (76.7)	
At least half the days	2 (7.1)	5 (16.7)	
Less than half the days	1 (3.6)	2 (6.7)	
Pain duration, *n* (%)			**.045**
3-6 months	0 (0.0)	3 (10.0)	
6-12 months	3 (10.7)	3 (10.0)	
1-5 years	14 (50.0)	6 (20.0)	
>5 years	11 (39.3)	18 (60.0)	
Treatment laterality, *n* (%)			.836^a^
Left	8 (28.6)	7 (23.3)	
Right	9 (32.1)	9 (30.0)	
Bilateral	11 (39.3)	14 (46.7)	
Treatment level, *n* (%)			.546
L2-L3	1 (3.6)	0 (0.0)	
L3-L4	3 (10.7)	1 (3.3)	
L4-L5	4 (14.3)	5 (16.7)	
L5-S1	20 (71.4)	24 (80.0)	
Listhesis present at lumbar levels, *n* (%)			.940^a^
No	18 (64.3)	19 (63.3)	
Yes	10 (35.7)	11 (36.7)	
Age, mean ± SD	56.6 ± 15.9	56.7 ± 14.9	.995^b^
BMI, mean ± SD	29.7 ± 5.9	30.9 ± 11.5	.632^b^
Baseline NPRS back pain score, mean ± SD	5.7 ± 1.9	5.9 ± 1.8	.754^b^
Baseline NPRS leg pain score, mean ± SD	6.6 ± 1.4	6.3 ± 1.1	.439^b^
Baseline ODI score, mean ± SD	33.2 ± 13.6	33.6 ± 11.7	.900^b^

Abbreviations: hAF, human amniotic fluid filtrate; SD, standard deviation.

*P* values are from Fisher’s exact test, unless otherwise specified. Bold values denote statistical significance at *P *< .05.

a From χ^2^ test.

b From *t*-test with unequal variance.

**Table 3:** Stenosis severity at treatment levels.

	Original treatment assignment		
Stenosis severity	hAF (*n *= 28)	Dexamethasone (*n *= 30)	*P*
Central			1.000
Normal	17/28 (60.7; 42.4-76.4)	18/30 (60.0; 42.3-75.4)	
Mild	9/28 (32.1; 17.9-50.7)	10/30 (33.3; 19.2-51.2)	
Moderate	2/28 (7.1; 2.0-22.7)	2/30 (6.7; 1.9-21.3)	
Severe	0/28 (0.0; 0.0-12.1)	0/30 (0.0; 0.0-11.4)	
Left foraminal			.714
Normal	6/17 (39.5; 17.3-58.7)	9/21 (42.9; 24.5-63.5)	
Mild	4/17 (23.5; 9.6-47.3)	7/21 (33.3; 17.2-54.6)	
Moderate	6/17 (35.3; 17.3-58.7)	4/21 (19.1; 7.7-40.0)	
Severe	1/17 (5.9; 1.1-27.0)	1/21 (4.8; 0.9-22.7)	
Right foraminal			.620
Normal	11/22 (50.0; 30.7-69.3)	8/23 (34.8; 18.8-55.1)	
Mild	4/22 (18.2; 7.3-38.5)	6/23 (26.1; 12.6-46.5)	
Moderate	5/22 (22.7; 10.1-43.4)	8/23 (34.8; 18.8-55.1)	
Severe	2/22 (9.1; 2.5-27.8)	1/23 (4.4; 0.8-21.0)	

Abbreviation: hAF, human amniotic fluid filtrate.

Data are presented as *n*/*N* participants (% with 95% CIs). *P* values are from Fisher’s exact test.

Results of blinding assessments gauging participants’ knowledge of their original treatment assignment are presented in Table 4. A majority of participants in each group reported that they were unsure of treatment assignment at both 3 weeks (69.6% hAF vs. 92.0% dexamethasone) and 6 months (71.4% hAF vs. 66.7% dexamethasone). These assessments of participants’ knowledge of treatment assignment, combined with largely balanced cohort characteristics, offer reassurance that blinding was maintained for the study duration.

**Table 4:** Treatment blinding assessment at 3 weeks and 6 months by treatment group.

	Original treatment assignment		
Suspected treatment assignment	hAF (*n *= 28)	Dexamethasone (*n *= 30)	*P*
3 weeks			.103
Definitely hAF	0/23 (0.0; 0.0-14.3)	0/25 (0.0; 0.0-13.3)	
Probably hAF	5/23 (21.7; 9.7-41.9)	1/25 (4.0; 0.7-19.5)	
I don’t know/unsure	16/23 (69.6; 49.1-84.4)	23/25 (92.0; 75.0-97.8)	
Probably dexamethasone	2/23 (8.7; 2.4-26.8)	1/25 (4.0; 0.7-19.5)	
Definitely dexamethasone	0/23 (0.0; 0.0-14.3)	0/25 (0.0; 0.0-13.3)	
6 months^a^			.383
Definitely hAF	0/21 (0.0; 0.0-15.5)	2/21 (9.5; 2.7-28.9)	
Probably hAF	4/21 (19.1; 7.7-40.0)	2/21 (9.5; 2.7-28.9)	
I don’t know/unsure	15/21 (71.4; 50.0-86.2)	14/21 (66.7; 45.4-82.8)	
Probably dexamethasone	1/21 (4.8; 0.9-22.7)	3/21 (14.3; 5.0-34.6)	
Definitely dexamethasone	1/21 (4.8; 0.9-22.7)	0/21 (0.0; 0.0-15.5)	

Abbreviation: hAF, human amniotic fluid filtrate.

Data are presented as *n*/*N* participants (% with 95% CIs). *P* values are from Fisher’s exact test.

a Outcomes at 6 months reflect effects of cross-over injection(s) in participants who elected to cross over to the alternative treatment at 3 months.

A summary of repeat and crossover injections for each treatment group is shown in Table 5. The proportion of participants who opted to receive a repeat injection according to their original treatment assignment within 3 months of randomization was significantly lower in the dexamethasone group compared to the hAF group (56.7% vs. 82.1%; *P *= .049). Similarly, a smaller proportion of participants originally assigned to the dexamethasone group elected to cross over and receive the alternative injection at 3 months (36.7% vs. 75.0%; *P *= .003). The dexamethasone group also had a lower repeat crossover injection rate compared to the hAF group, but this difference did not achieve statistical significance (26.7% vs. 42.9%; *P *= .195).

**Table 5:** Additional and crossover injections by treatment group.

	Original treatment assignment		
Additional injection	hAF (*n *= 28)	Dexamethasone (*n *= 30)	*P*
Continuation injection within 3 months of randomization	23/28 (82.1; 64.4-92.2)	17/30 (56.7; 39.2-72.6)	**.049**
Crossover to alternative injection at 3 months	21/28 (75.0; 56.6-87.3)	11/30 (36.7; 21.9-54.5)	**.003** ^a^
Repeat of crossover injection	12/28 (42.9; 26.5-60.9)	8/30 (26.7; 14.2-44.5)	.195^a^

Abbreviation: hAF, human amniotic fluid filtrate.

Data are presented as *n*/*N* participants (% with 95% CIs). *P* values are from Fisher’s exact test, unless otherwise specified. Bold values denote statistical significance at *P *< .05.

a From x^2^ test.

### Responder analyses

The proportion of participants with ≥50% NPRS improvement in back pain was consistently higher at all follow-up timepoints for the dexamethasone group compared to the hAF group (Table 6). The between-group difference was statistically significant at 6 weeks, with a dexamethasone group responder rate of 54.2% (95% CI: 35.1%-72.1%) compared to only 4.6% (95% CI: 0.8%-21.8%) in the hAF group (*P *< .001). Response rates for ≥50% NPRS leg pain improvement were more similar between groups than those for back pain. However, the proportion of participants reporting this outcome was significantly greater at 3 months in the dexamethasone versus hAF group (60.0% [95% CI: 40.7%-76.6%] vs. 25.0% [95% CI: 11.2%-46.9%]; *P *= .034).

**Table 6:** Responder rates for back and leg pain improvement by follow-up timepoint.

	Original treatment assignment		
Outcome and timepoint	hAF (*n *= 28)	Dexamethasone (*n *= 30)	*P*
≥50% NPRS back pain improvement			
3 weeks	3/23 (13.0; 4.5-32.1)	8/24 (33.3; 18.0-53.3)	.168
**6 weeks**	**1/22 (4.6; 0.8**-**21.8)**	**13/24 (54.2; 35.1**-**72.1)**	**<.001**
3 months	3/20 (15.0; 5.2-36.0)	11/24 (45.8; 27.9-64.9)	.050
6 months^a^	8/21 (38.1; 20.8-59.1)	10/20 (50.0; 29.9-70.1)	.536
≥50% NPRS leg pain improvement			
3 weeks	7/23 (30.4; 15.6-50.9)	9/25 (36.0; 20.3-55.5)	.765
6 weeks	8/22 (36.4; 19.7-57.1)	11/25 (44.0; 26.7-62.9)	.767
**3 months**	**5/20 (25.0; 11.2**-**46.9)**	**15/25 (60.0; 40.7**-**76.6)**	**.034**
6 months^a^	12/21 (57.1; 36.6-75.5)	12/21 (57.1; 36.6-75.5)	1.000

Abbreviations: hAF, human amniotic fluid filtrate; NPRS, numerical pain rating scale.

Data are presented as *n*/*N* participants (% with 95% CIs). *P* values are from Fisher’s exact test. Bold values denote statistical significance at *P *< .05.

a Outcomes at 6 months reflect effects of cross-over injection(s) in participants who elected to cross over to the alternative treatment at 3 months.

Responder rates for ≥30% ODI improvement were generally higher for the dexamethasone group compared to the hAF group until 6 months, when there was a reversal of this trend after the elective 3-month cross-over timepoint (Table 7). No between-group differences achieved statistical significance at any follow-up timepoint (*P *> .05). Similarly, PROMIS PHS and MHS responder rates (defined as ≥5-point improvement from baseline) showed no consistent patterns favoring either treatment group, and no between-group differences were statistically significant at any follow-up timepoint (Table 8; *P *> .05).

**Table 7:** Responder rates for Oswestry Disability Index by follow-up timepoint.

	Original treatment assignment		
Outcome and timepoint	hAF (*n *= 28)	Dexamethasone (*n *= 30)	*P*
≥30% ODI improvement			
3 weeks	6/23 (26.1; 12.6-46.5)	10/25 (40.0; 23.4-59.3)	.368
6 weeks	2/22 (9.1; 2.5-27.8)	5/25 (20.0; 8.9-39.1)	.423
3 months	1/20 (5.0; 0.9-23.6)	5/25 (20.0; 8.9-39.1)	.205
6 months^a^	7/21 (33.3; 17.2-54.6)	5/21 (23.8; 10.6-45.1)	.734

Abbreviations: hAF, human amniotic fluid filtrate; ODI, Oswestry Disability Index.

Data are presented as *n*/*N* participants (% with 95% CIs). *P* values are from Fisher’s exact test.

a Outcomes at 6 months reflect effects of cross-over injection(s) in participants who elected to cross over to the alternative treatment at 3 months.

**Table 8:** Responder rates for PROMIS by follow-up timepoint.

	Original treatment assignment		
Outcome and timepoint	hAF (*n *= 28)	Dexamethasone (*n *= 30)	*P*
≥5-pt PROMIS PHS improvement			
3 weeks	9/21 (42.9; 24.5-63.5)	5/23 (21.7; 9.7-41.9)	.133^a^
6 weeks	6/22 (27.3; 13.2-48.2)	10/25 (40.0; 23.4-59.3)	.358^a^
3 months	4/20 (20.0; 8.1-41.6)	10/25 (40.0; 23.4-59.3)	.202
6 months^b^	10/20 (50.0; 29.9-70.1)	8/21 (38.1; 20.8-59.1)	.443^a^
≥5-pt PROMIS MHS improvement			
3 weeks	3/22 (13.6; 4.8-33.3)	4/25 (16.0; 6.4-34.7)	1.000
6 weeks	4/22 (18.2; 7.3-38.5)	3/25 (12.0; 4.2-30.0)	.690
3 months	1/20 (5.0; 0.9-23.6)	5/25 (20.0; 8.9-39.1)	.205
6 months^b^	4/21 (19.1; 7.7-40.0)	6/21 (28.6; 13.8-50.0)	.719

Abbreviations: hAF, human amniotic fluid filtrate; MHS, mental health summary; PHS, physical health summary; PROMIS, Patient Reported Outcomes Measurement Information System.

Data are presented as *n*/*N* participants (% with 95% CIs). *P* values are from Fisher’s exact test, unless otherwise specified.

a From χ^2^ test.

b Outcomes at 6 months reflect effects of cross-over injection(s) in participants who elected to cross over to the alternative treatment at 3 months.

Outcomes for SSSQ symptom severity (subdivided into pain and neuroischemic domains) are summarized in Table 9. Dexamethasone group responder rates for ≥30% SSSQ improvement were higher at all timepoints compared to the hAF group for overall symptom severity and pain. However, the only between-group difference to achieve statistical significance was for pain subscale responder analysis at 6 weeks (44.0% [95% CI: 26.7%-62.9%] dexamethasone vs. 4.6% [95% CI: 0.8%-21.8%] hAF; *P *= .002). Neuroischemic subscale outcomes were more consistent between groups, with no significant differences at any timepoint.

**Table 9:** Responder rates for Swiss Spinal Stenosis Questionnaire by follow-up timepoint.

	Original treatment assignment		
Outcome and timepoint	hAF (*n *= 28)	Dexamethasone (*n *= 30)	*P*
≥30% SSSQ symptom severity improvement			
3 weeks	3/23 (13.0; 4.5-32.1)	7/25 (28.0; 14.3-47.6)	.292
6 weeks	1/22 (4.6; 0.8-21.8)	6/25 (24.0; 11.5-43.4)	.102
3 months	2/20 (10.0; 2.8-30.1)	9/25 (36.0; 20.3-55.5)	.079
6 months^b^	7/21 (33.3; 17.2-54.6)	9/21 (42.9; 24.5-63.5)	.525^a^
≥30% SSSQ pain improvement			
3 weeks	5/23 (21.7; 9.7-41.9)	8/25 (32.0; 17.2-51.6)	.523
**6 weeks**	**1/22 (4.6; 0.8**-**21.8)**	**11/25 (44.0; 26.7**-**62.9)**	**.002**
3 months	4/20 (20.0; 8.1-41.6)	10/25 (40.0; 23.4-59.3)	.202
6 months^b^	9/21 (42.9; 24.5-63.5)	11/21 (52.4; 32.4-71.7)	.537^a^
≥30% SSSQ neuroischemic improvement			
3 weeks	4/23 (17.4; 7.0-37.1)	7/25 (28.0; 14.3-47.6)	.499
6 weeks	8/22 (36.4; 19.7-57.1)	5/25 (20.0; 8.9-39.1)	.328
3 months	5/20 (25.0; 11.2-46.9)	6/25 (24.0; 11.5-43.4)	1.000
6 months^b^	6/21 (28.6; 13.8-50.0)	5/21 (23.8; 10.6-45.1)	1.000

Abbreviations: hAF, human amniotic fluid filtrate; SSSQ, Swiss Spinal Stenosis Questionnaire.

Data are presented as *n*/*N* participants (% with 95% CIs). *P* values are from Fisher’s exact test, unless otherwise specified. Bold values denote statistical significance at *P *< .05.

a From χ^2^ test.

b Outcomes at 6 months reflect effects of cross-over injection(s) in participants who elected to cross over to the alternative treatment at 3 months.

The proportion of participants who reported being “better” or “a great deal better” with scores ≥5 on PGIC was consistently higher at all timepoints for the dexamethasone versus hAF group (Table 10). However, no between-group differences demonstrated statistical significance (*P *> .05).

**Table 10:** Responder rates for Patient Global Impression of Change by follow-up timepoint.

	Original treatment assignment		
Outcome and timepoint	hAF (*n *= 28)	Dexamethasone (*n *= 30)	*P*
≥5 PGIC^b^			
3 weeks	1/23 (4.4; 0.8-21.0)	7/25 (28.0; 9.0-14.3)	.050^a^
6 weeks	3/22 (13.6; 4.8-33.3)	8/25 (32.0; 17.2-51.6)	.138
3 months	3/20 (15.0; 5.2-36.0)	9/25 (36.0; 20.3-55.5)	.113
6 months^c^	7/21 (33.3; 17.2-54.6)	11/21 (52.4; 32.4-71.7)	.212

Abbreviations: CI, confidence interval; hAF, human amniotic fluid filtrate; PGIC, Patient Global Impression of Change.

Data are presented as *n*/*N* participants (% with 95% CIs). *P* values are from χ^2^ test, unless otherwise specified.

a From Fisher’s exact test.

b PGIC scores ≥ 5 indicate “better” or “a great deal better.”

c Outcomes at 6 months reflect effects of cross-over injection(s) in participants who elected to cross over to the alternative treatment at 3 months.

### Linear regression analyses of patient-reported outcomes

**Figure 2:**
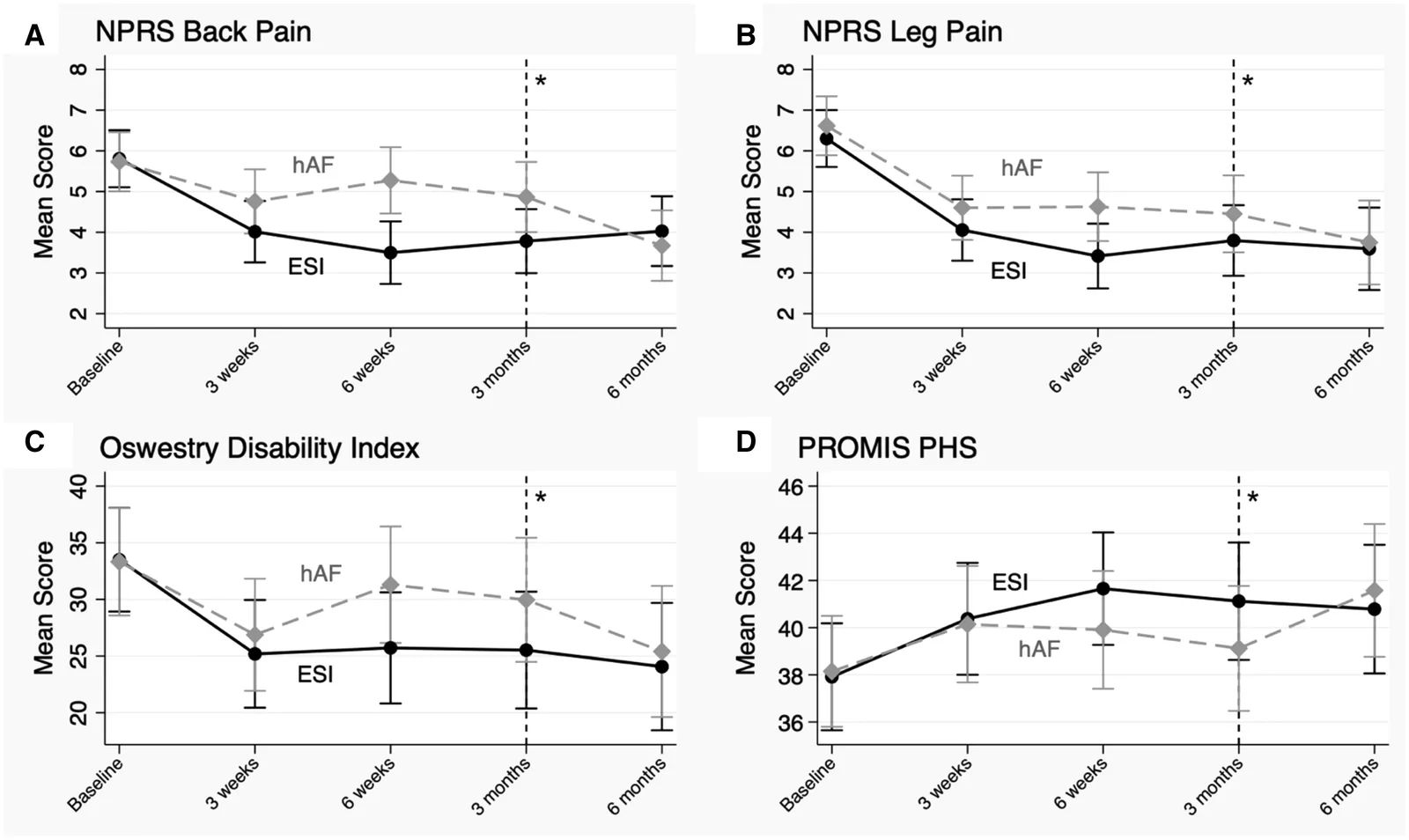
Adjusted mean scores for (A) back and (B) leg numerical pain rating scale (NPRS), (C) Oswestry Disability Index, and (D) PROMIS Physical Health Summary by treatment group over time calculated from regression models, setting the covariate for pain duration to the mean distribution. ^*^Dashed vertical lines indicate the optional cross-over timepoint at 3 months. As such, outcomes at 6 months reflect effects of the cross-over injection(s) in participants who elected to receive the alternative treatment.

**Figure 3:**
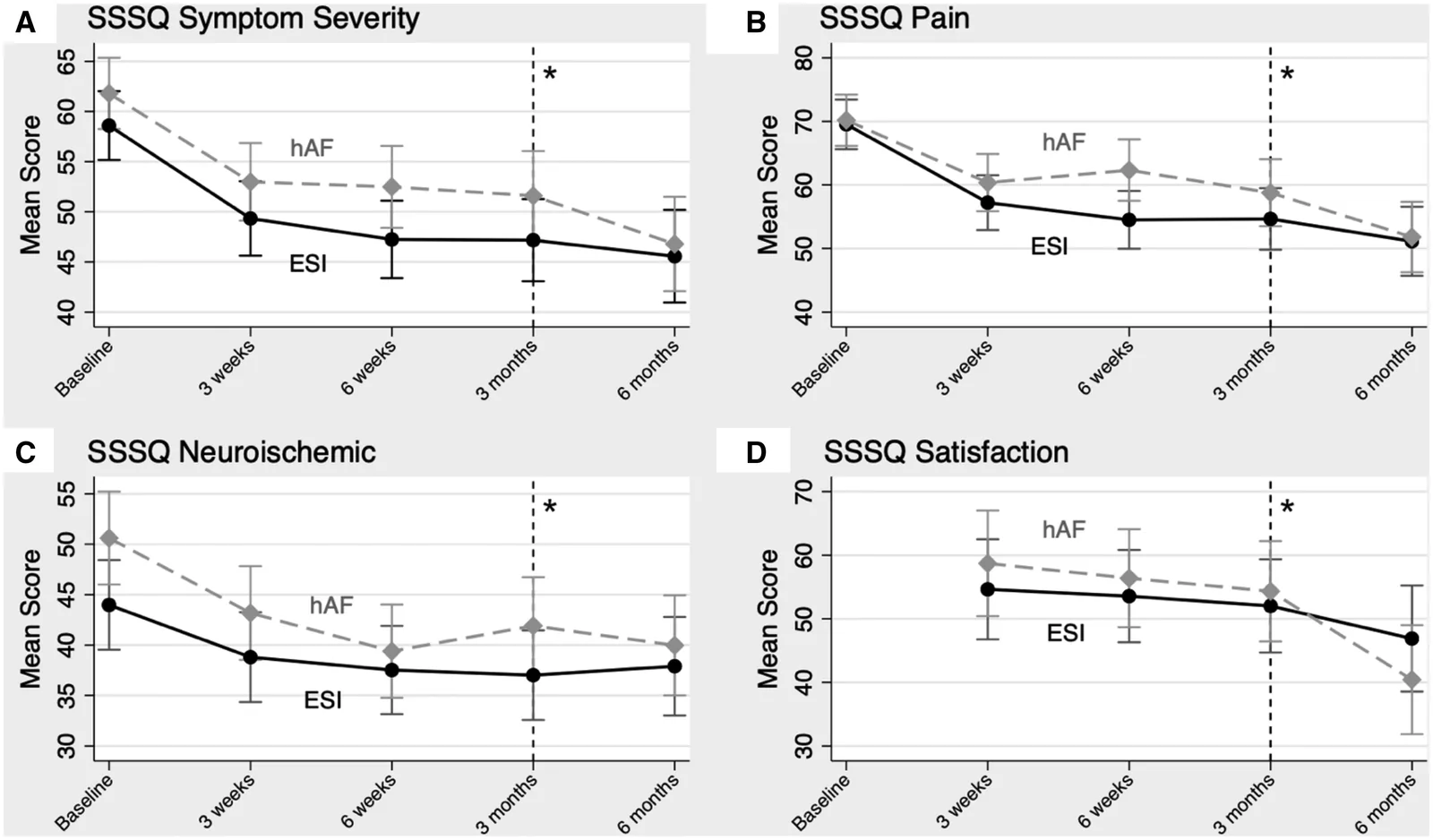
Adjusted mean scores for (A) symptom severity, (B) pain, (C) neuroischemic, and (D) satisfaction subscales on Swiss Spinal Stenosis Questionnaire (SSSQ) by treatment group over time calculated from regression models, setting the covariate for pain duration to the mean distribution. ^*^Dashed vertical lines indicate the optional cross-over timepoint at 3 months. As such, outcomes at 6 months reflect effects of the cross-over injection(s) in participants who elected to receive the alternative treatment.

Regression-adjusted mean scores for NPRS (back and leg pain), ODI, PROMIS PHS, and SSSQ (severity, pain, neuroischemic, and satisfaction subscales) by treatment group over time are displayed in Figures 2 and 3. Multivariable linear regression modelling revealed that mean dexamethasone group scores for NPRS back pain, NPRS leg pain, ODI, and PROMIS PHS, but not PROMIS MHS, were significantly improved from baseline at all follow-up timepoints after adjusting for pain duration, which differed between groups despite randomization (Table 11; *P *< .05). A significant hAF x timepoint interaction term in the models for NPRS back pain and ODI indicated that back pain (β = 1.85; *P *= .002) and functional (β = 5.76; *P *= .023) scores improved to a greater extent in the dexamethasone group compared to hAF at 6 weeks.

**Table 11:** Multivariable longitudinal linear regression models on hAF vs. dexamethasone treatment effect differences in mean NPRS, ODI, and PROMIS scores over time.

	NPRS (back)		NPRS (leg)		ODI		PROMIS PHS		PROMIS MHS	
Covariates	β	*P*	β	*P*	β	*P*	β	*P*	β	*P*
Treatment (vs. dexamethasone)										
hAF	−0.47	.377	0.07	.896	−1.11	.756	0.94	.596	0.43	.858
Timepoint (vs. baseline)										
3 weeks	−1.79	<.001	−2.25	<.001	−8.31	<.001	2.46	.006	−1.06	.285
6 weeks	−2.31	<.001	−2.89	<.001	−7.79	<.001	3.74	<.001	−0.54	.599
3 months	−2.02	<.001	−2.50	<.001	−7.98	<.001	3.20	.002	0.71	.517
6 months	−1.78	<.001	−2.71	<.001	−9.43	<.001	2.87	.016	1.74	.163
hAF x timepoint interaction										
3 weeks	0.82	.172	0.23	.693	1.87	.428	−0.46	.721	−0.28	.848
6 weeks	**1.85**	**.002**	0.90	.154	**5.76**	**.023**	−1.98	.142	−2.38	.114
3 months	1.16	.066	0.34	.625	4.62	.102	−2.23	.137	−2.28	.162
6 months	−0.28	.669	−0.16	.838	1.52	.634	0.56	.742	−2.77	.122
Pain duration (vs. 3-6 months)										
6-12 months	2.49	.020	−0.24	.831	3.16	.714	−7.65	.064	−1.16	.843
1-5 years	3.03	.002	0.66	.511	6.23	.423	−5.57	.134	−2.09	.691
> 5 years	2.93	.001	−0.20	.836	5.81	.431	−4.63	.189	0.09	.985

Abbreviations: hAF, human amniotic fluid filtrate; MHS, mental health summary; NPRS, numerical pain rating scale; ODI, Oswestry Disability Index; PHS, physical health summary; PROMIS, Patient Reported Outcomes Measurement Information System.

Bold values denote treatment effect differences achieving statistical significance at *P *< .05.

In the dexamethasone group, mean SSSQ scores for the severity, pain, and neuroischemic subscales were significantly reduced from baseline (indicating improvements to back and limb symptoms) at all follow-up timepoints after adjusting for pain duration (Table 12; *P *< .05). However, mean SSSQ satisfaction scores did not change significantly from the 3-week reference value at any subsequent timepoint (*P *> .05). Treatment effects were statistically similar between groups with the exception of 6-week outcomes for pain on SSSQ, which were significantly better in the dexamethasone group compared to hAF (β = 7.17; *P *= .030).

**Table 12:** Multivariable longitudinal linear regression models on hAF vs. dexamethasone treatment effect differences in mean Swiss Spinal Stenosis Questionnaire scores over time.

	Severity		Pain		Neuroischemic		Satisfaction	
Covariates	β	*P*	β	*P*	β	*P*	β	*P*
Treatment (vs. dexamethasone)								
hAF	1.89	.473	−0.10	.974	4.49	.187	1.69	.782
Timepoint (vs. baseline)								
3 weeks	−9.27	<.001	−12.32	<.001	−5.18	.012	(ref)	—
6 weeks	−11.36	<.001	−15.02	<.001	−6.46	.003	−1.08	.723
3 months	−11.43	<.001	−14.88	<.001	−6.97	.003	−2.62	.462
6 months	−13.04	<.001	−18.40	<.001	−6.08	.023	−7.75	.085
hAF x timepoint interaction								
3 weeks	0.45	.869	2.50	.433	−2.25	.450	(ref)	—
6 weeks	2.04	.476	**7.17**	**.030**	−4.76	.131	−1.26	.776
3 months	1.23	.690	3.47	.321	−1.75	.614	−1.77	.735
6 months	−1.98	.554	0.02	.996	−4.56	.231	−10.55	.102
Pain duration (vs. 3-6 months)								
6-12 months	1.50	.792	−1.19	.857	7.60	.274	−6.10	.630
1-5 years	5.65	.271	1.44	.810	13.03	.036	5.45	.630
>5 years	2.59	.594	−1.70	.763	10.64	.070	−2.58	.810

Abbreviation: hAF, human amniotic fluid filtrate.

Bold values denote treatment effect differences achieving statistical significance at *P *< .05.

### Adverse events

A total of five Serious Adverse Events (SAEs) occurred in four participants. None of these events were determined to be related to the study intervention. A total of 148 adverse events (AEs) of any severity or relatedness were reported, with 90 AEs occurring in 18 dexamethasone group participants and 58 AEs among 22 hAF group participants. There were no between-group differences in AE severity (mild, moderate, severe, or involving death; *P *= .229), relatedness to the study intervention (not, probably not, possibly, or probably related; *P *= .974), or the distribution of unexpected (vs. expected) AEs (*P *= .669; Table 13). The three most commonly reported AEs in both groups were exacerbation of typical back/leg pain (*n *= 11/28 or 32.1% for the hAF group vs. *n *= 11/30 or 36.7% for the dexamethasone group), subjective report of new worsening leg weakness (*n *= 7/28 or 25.0% for hAF vs. *n *= 5/30 or 16.7% for dexamethasone), and headaches (*n *= 7/28 or 25.0% for hAF vs. *n *= 5/30 or 16.7% for dexamethasone). The next most common AE among hAF group participants was nausea (*n *= 5/28; 17.9%), followed by insomnia (*n *= 3/28; 10.7%) and anxiety (*n *= 3/28; 10.7%). Insomnia was also among the top five AEs reported by dexamethasone group participants (*n *= 4/30; 13.3%), along with paresthesia/dysesthesia of the lower back and/or limbs (*n *= 5/30; 16.7%).

**Table 13:** Number of adverse events by treatment group.

	Original treatment assignment		
AE classification	hAF (*n *= 58 AEs)	Dexamethasone (*n *= 90 AEs)	*P*
Severity			.202^a^
Mild	43/58 (74.1; 61.6-83.7)	54/90 (60.0; 49.7-69.5)	
Moderate	13/58 (22.4; 13.6-34.7)	26/90 (28.9; 20.5-39.0)	
Severe	2/58 (3.5; 1.0-11.7)	9/90 (10.0; 5.4-17.9)	
Involved death	0/58 (0.0; 0.0-6.2)	1/90 (1.1; 0.2-6.0)	
Relationship to study intervention			.974
Not related	25/58 (43.1; 31.2-55.9)	42/90 (46.7; 36.7-56.9)	
Probably not related	13/58 (22.4; 13.6-34.7)	18/90 (20.0; 13.0-29.4)	
Possibly related	9/58 (15.5; 8.4-26.9)	13/90 (14.4; 8.6-23.2)	
Probably related	11/58 (19.0; 10.9-30.9)	17/90 (18.9; 12.1-28.2)	
Expected			.669
Yes	20/58 (34.5; 23.6-47.3)	28/90 (31.1; 22.5-41.3)	
No	38/58 (65.5; 52.7-76.4)	62/90 (68.9; 58.7-77.5)	

Abbreviations: AE, adverse event; hAF, human amniotic fluid filtrate.

Data are presented as *n*/*N* adverse events (% with 95% CIs). *P* values are from χ^2^ test, unless otherwise specified.

a From Fisher’s exact test.

The mean number of AEs per participant did not significantly differ between groups (2.1 ± 1.8 for hAF vs. 3.0 ± 4.0 for dexamethasone; *P *= .251). Similarly, the mean number of possibly or probably related AEs did not differ between groups (1.5 ± 1.8 for hAF vs. 1.5 ± 2.5 for dexamethasone; *P *= .995). However, the mean number of unexpected (vs. expected) AEs per participant was significantly greater in the dexamethasone group (4.3 ± 3.3) compared to the hAF group (2.1 ± 0.9; *P *= .043).

The proportion of participants who reported any type of AE did not significantly differ between hAF (*n *= 22/28; 79.0%) and dexamethasone (*n *= 18/30; 60.0%) treatment groups (*P *= .127; Table 14). There was no between-group difference in the proportion of participants who experienced AEs that were rated as severe or involved death: 7.1% (*n *= 2/28) for hAF versus 16.7% (*n *= 5/30) for dexamethasone (*P *= .425). Lastly, there was no difference between groups in the proportion of participants who experienced AEs that were probably or definitely related to the study intervention: 50.0% (*n *= 14/28) for hAF versus 70.0% (*n *= 21/30) for dexamethasone (*P *= .120).

**Table 14:** Proportions of participants with adverse events by treatment group.

	Original treatment assignment		
AE classification	hAF (*n *= 28)	Dexamethasone (*n *= 30)	*P*
Any AE	22/28 (78.6; 60.5-89.8)	18/30 (60.0; 42.3-75.4)	.127
Rated as severe	2/28 (7.1; 2.0-22.7)	4/30 (13.3; 5.3-29.7)	.671^a^
Probably or definitely related to study intervention	14/28 (50.0; 32.6-67.4)	21/30 (70.0; 32.6-83.3)	.120

Abbreviations: AE, adverse event; hAF, human amniotic fluid filtrate.

Data are presented as *n*/*N* participants (% with 95% CIs). *P* values are from χ^2^ test, unless otherwise specified.

a From Fisher’s exact test.

## Discussion

This is the first study to evaluate the safety and effectiveness of transforaminal epidural injection with sterile hAF in comparison to dexamethasone for the treatment of lumbosacral radicular pain due to spinal stenosis. In this double-blinded, prospective randomized trial, we observed that while hAF demonstrated a favorable safety profile, transforaminal epidural dexamethasone produced superior pain relief. This led to early termination of the study based on hAF treatment futility. The superior pain relief observed with dexamethasone compared to hAF may be attributed to several factors, including the complexity of the inflammatory pathways involved in spinal stenosis and radicular pain,^44^ as well as potential differences in the potency of anti-inflammatory effect of dexamethasone compared to hAF at the relative concentrations used in the present study. Though amniotic fluid has shown promise in regenerative medicine for tissue repair,^45^ its effects on pain modulation, reduction of inflammation, and neural protection in the context of spinal stenosis remain unclear and may be less robust than those of corticosteroids such as dexamethasone.

While the present study demonstrated minimal treatment effect associated with transforaminal epidural hAF, we observed a strong treatment effect following transforaminal epidural injection of dexamethasone. This is notable given that few well-controlled studies have evaluated TFESI for LSS. In a large multicenter RCT, Friedly et al found TFESI with lidocaine to be no more effective than epidural lidocaine alone at 3 or 6 weeks for central canal lumbar spinal stenosis.^26^ Epidural lidocaine demonstrates a treatment effect,^46^^,^^47^ so the findings of Friedly et al suggest that adding corticosteroid to lidocaine in an epidural injection may not be necessary for treatment benefit. In the present study, we did not mix lidocaine with dexamethasone in the corticosteroid epidural injection group but interestingly observed a significant treatment effect without the presence of lidocaine. A three-arm study in which (1) dexamethasone alone, (2) lidocaine alone, and (3) dexamethasone combined with lidocaine would elucidate the relative treatment effect of each of these components during epidural injection for the treatment of radicular pain due to LSS.

A 2020 review by Smith et al found low-quality evidence for TFESI as a treatment for LSS-related radicular pain, noting that observational data were promising but required further corroboration from RCTs.^11^ Using a definition of ≥50% pain reduction, pooled success rates for TFESI across the available literature were 49% (range: 43%-55%) at 1 month, 48% (35%-61%) at 3 months, and 43% (33%-53%) at 6 months. Our results are well-aligned with these calculations: in the dexamethasone treatment group, 36%, 44%, 60%, and 57% of participants reported ≥50% reductions in radicular pain on NPRS at 3 weeks, 6 weeks, 3 months, and 6 months, respectively. Notably, our study sample was comprised of participants who largely had mild to moderate neuroforaminal stenosis as the cause of their radicular pain symptoms (as opposed to central canal stenosis). These findings contribute to the body of evidence supporting TFESI for the treatment of lumbosacral radicular pain due to LSS.

To date, only one other study has investigated amniotic fluid as an anti-inflammatory treatment agent for low back pain due to lumbar spinal stenosis.^48^ Buttermann et al recently published results of a prospective pilot study comparing outcomes of transforaminal epidural injection with amniotic fluid in three groups of patients whose axial LBP (with or without leg symptoms) was primarily attributed to lumbar herniated nucleus pulposus (HNP), LSS, and degenerative disc disease (DDD), respectively.^48^ Compared to LSS and DDD, patients in the HNP group experienced the greatest improvements to back and leg pain, ODI, and PROMIS physical component scores over the 1-year follow-up duration. Amniotic fluid demonstrated moderate effectiveness in patients with LSS, and was least effective for DDD patients. In comparison with our 3-month results, LSS group responder rates at 3-4 months were substantially higher: approximately 60% reported ≥50% reductions in both back and leg pain (vs. 15% and 25% in our hAF cohort), while around 40% achieved ≥30% ODI improvement (vs. 5%). However, this disparity diminished at later follow-up timepoints. At 6-8 months, LSS group responder rates for ≥50% pain reduction were approximately 50% for both back and leg pain, which closely aligned with our 6-month hAF group results (38% and 57%, respectively). ODI responder rates were also comparable, with 35% of LSS patients and 33% of our hAF group achieving ≥30% improvement. The improvements to 6-month responder rates for pain and function in our study are likely due to cross-over at 3 months post-randomization, when 75% of participants originally allocated to the hAF group opted to receive the alternative injection with dexamethasone. The high initial responder rates observed by Buttermann et al through 3-4 months may also have been influenced by steroid treatment effects, as the authors reported that 65% of patients in their LSS group had undergone ESI prior to receiving hAF.

Minimal comparable research has been published on the use of amniotic fluid and membrane-based injections for spinal pain indications. Miedema et al^49^ evaluated outcomes of transforaminal epidural injection of amniotic membrane and umbilical cord (AMUC) particulate in a case series of 12 patients with lumbar radiculopathy. The authors reported a significant reduction in pain scores, with the average scores decreasing from 6.6 ± 1.5 at baseline to 5.2 ± 1.9 at 1-3 months, 2.0 ± 1.4 at 6 months, and 2.9 ± 1.4 at the last mean follow-up of approximately 21 months. Buck investigated the effect of intradiscal injections of AMUC particulate in 11 patients with discogenic low back pain.^50^ The author reported median pain reductions of 40% at 1 month (*n *= 6), 50% at 3 months (*n *= 8), and 75% at 6 months (*n *= 5), with no adverse events or complications observed during the 6-month follow-up period. While these early studies suggest feasibility of amniotic fluid or membrane-based therapies for spinal pain indications, additional research is needed to better understand the potential safety, indications, efficacy, and effectiveness of these types of agents.

### Limitations

This study has limitations that must be acknowledged. As a single-center randomized trial, generalizability and external validity of our results may be constrained. Premature termination of the study as recommended by the DSMB resulted in a smaller sample size and shorter follow-up period than intended. Outcomes were only collected through 6 months, which restricts our ability to assess the long-term effects and durability of hAF compared to dexamethasone TFESI in those participants who did not cross-over. The smaller sample size limited power to detect potential factors that may have predicted treatment response. This study population primarily consisted of patients with mild to moderate foraminal stenosis, with relatively few cases of central canal stenosis. Accordingly, the findings may not be generalizable to patients with severe central stenosis. Although we found no apparent disparities in adverse event rates between the hAF and dexamethasone groups, it is possible that differences in safety outcomes could have emerged at extended follow-up with a larger sample. Furthermore, research on the cardioprotective effects of hAF in animal models suggests that the total protein content (approximately 3.0-8.4 mg^51^) remaining in 3 mL of hAF after processing and filtration is below the minimal effective threshold for localized injection,^52^^,^^53^ introducing the possibility that we underdosed patients in the hAF group. Future study with this particular agent should take into consideration that higher doses of the therapeutic constituents within the hAF may be required.

### Future research

Future research should explore the optimal use of hAF, possibly in different concentrations/volumes, as well as for different spinal pain indications, such as symptomatic facet joint arthropathy, discogenic low back pain, vertebrogenic low back pain, and lumbar radicular pain due to disc herniation (rather than LSS). Safety and treatment effects in larger study samples, along with comparisons to sham injection and usual care, are among the many relevant research questions that must be answered to understand whether hAF has therapeutic value for radicular or other spinal pain indications.

## Conclusions

We report outcomes from the first prospective randomized trial comparing transforaminal epidural injection of hAF to dexamethasone for the treatment of radicular pain associated with lumbosacral spinal stenosis. While success rates were fairly similar between groups, pain relief with dexamethasone was superior to hAF at several timepoints, including ≥50% NPRS back pain reduction (54% [95% CI: 35%-72%] vs. 5% [95% CI: 1%-22%]) and ≥30% SSSQ pain subscale improvement (44% [95% CI: 27%-63%] vs. 5% [95% CI: 1%-22%]) at 6 weeks and ≥50% NPRS leg pain reduction at 3 months (60% [95% CI: 41%-77%] vs. 25% [95% CI: 11%-47%]). These findings were corroborated by regression modeling, which showed consistent treatment benefits of dexamethasone through the primary endpoint of 3 months, with pain and functional scores improving to a greater extent at 6 weeks in the dexamethasone group compared to hAF. The safety profiles of hAF and dexamethasone appeared equivalent: adverse event rates did not differ between groups, and there were no study-related serious adverse events. Although we found no support for hAF in managing radicular pain due to LSS, our results contribute to the existing evidence base for transforaminal epidural steroid injection as a safe and effective treatment for this condition.

## Supplementary Material

pnag048_Supplementary_Data
