# Supplementary material for: The safety and effectiveness of lumbar transforaminal injection of sterile amniotic fluid filtrate compared to steroid for lumbosacral radicular pain due to spinal stenosis: a phase I/II double-blinded randomized trial
Source: Pain Med. 2026 Mar 31;27(9):972–88. doi: 10.1093/pm/pnag048 (PMC13537739; doi:10.1093/pm/pnag048)
Supplement: Supplementary file 1 [file pnag048_supplementary_data.docx]

**Table S1.** Classification of adverse events

| **Severity** | **Serious Adverse Events** | **Unexpected Adverse Events** | **Expected Adverse Events** |
| --- | --- | --- | --- |
| - Mild: Requires minimal or no treatment and does not interfere with the participant’s daily activities. - Moderate: Results in a low level of inconvenience or concern with the therapeutic measures. Moderate events may cause some interference with functioning. - Severe: Interrupts a participant’s usual daily activity and may require systemic drug therapy or other treatment. Severe events are usually potentially life-threatening or incapacitating. | - Death - New life-threatening problem - An emergency department visit - Any hospitalization - Newly disabled or incapacitated - Required intervention to prevent permanent impairment - Other new “serious” medical event or problem | - Any new, unexpected, or untoward medical problem - Unexpected visits with a doctor or other health care specialist - Any new labs or imaging tests ordered or performed that were not expected | - Headache, facial flushing, nausea - Exacerbation of typical back and/or leg pain - Insomnia - Sensation of heart palpitations - Anxiety - New worsening leg weakness |
